# Effect of Process Parameters on HNO_*y*=2,3_ and NO_*y*_^–^ Formation in Plasma-Treated Water

**DOI:** 10.1021/acsomega.5c07020

**Published:** 2025-12-02

**Authors:** Jin Hee Bae, Seong-Cheol Huh, Negar Rahdar, Hyungyu Lee, Sanghoo Park

**Affiliations:** † Department of Nuclear and Quantum Engineering, 163715 Korea Advanced Institute of Science and Technology, 291 Daehak-Ro, Yuseong-Gu, Daejeon 34141, Republic of Korea; ‡ Department of Electronic and Biological Physics, 34968 Kwangwoon University, 20 Gwangun-Ro, Nowon-Gu, Seoul 01897, Republic of Korea

## Abstract

Here, we investigate the effects of hydrodynamics and temperature on the formation of nitrous and nitric acids (HNO_*y*=2,3_) and their conjugate bases (NO_*y*_^–^) in plasma-treated water (PTW) in a surface dielectric barrier discharge (sDBD) reactor. The stirring rate and bulk water temperature were systematically varied from 200 to 1000 rpm and 20 to 80 °C, respectively, while the plasma was held constant. Species in both the gas and liquid phases were tracked in real time using synchronized dual optical absorption spectroscopy, allowing correlation of the gas-phase O_3_/NO_*x*=1–3_ dynamics with the evolving aqueous species of NO_2_^–^, HNO_2_, and NO_3_^–^. Nitrate production monotonically accelerated with increasing agitation and dominated above 800 rpm, whereas the nitrite pool, NO_2_^–^ + HNO_2_, peaked at a moderate speed of 600 rpm and declined at higher speeds, probably due to volatilization losses. Elevated water temperature suppressed NO and NO_2_ formation, extended the O_3_-rich regime, and shifted selectivity toward NO_3_^–^; conversely, cooler conditions favored nitrite accumulation. These trends reveal the temperature-dependent kinetics and mass-transfer limits that govern plasma-driven nitrogen fixation. Our findings offer practical guidelines for tuning the composition of PTW, thereby advancing its efficient, application-specific production at scale.

## Graphical abstract

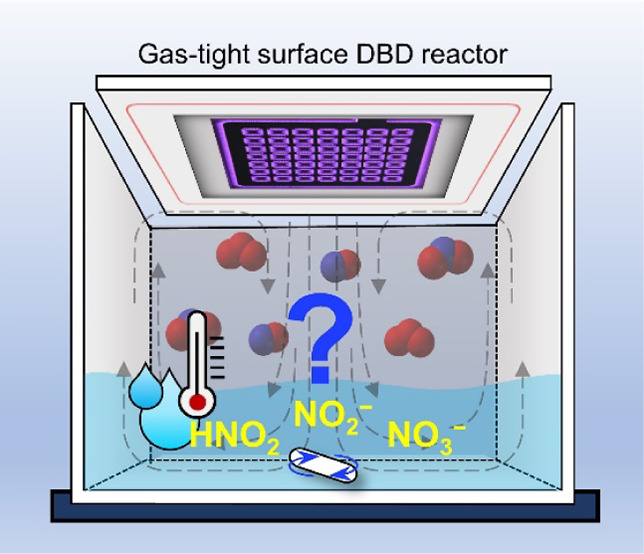

## Introduction

Nitrogen is an essential element for biological growth and survival.^1^ It forms the backbone of many biological macromolecules and governs key metabolic and energy-related processes in living organisms.^2,3^ Modern industry likewise heavily depends on fixed nitrogen compounds. Because nitrogen molecules (N_2_) in nature (i.e., the atmosphere) are chemically inert due to the strong triple bond, most organisms and processes cannot directly use atmospheric nitrogen. Instead, nitrogen must undergo a fixation process that converts it into bioavailable or industrially processable forms such as ammonia or nitrate.

At the industrial scale, ammonia synthesis through nitrogen fixation relies exclusively on the Haber–Bosch process, which accounts for ≥99% of global production.^4^ This single technique, however, consumes 1–2% of the global energy supply and is responsible for significant carbon emissions.^5^ The centralized, energy-intensive infrastructure required by the Haber–Bosch process also limits access to on-demand or small-scale applications. These drawbacks have stimulated worldwide efforts to develop alternative nitrogen-fixation routes.

Plasma-based nitrogen fixation has (re)­emerged as a compelling candidate. Low-temperature plasma, which is a nonequilibrium, reactive ionized gas generated by electricity, can activate atmospheric N_2_ and O_2_ and can be utilized for relevant chemical processes, even under ambient conditions; catalysts are optional, eliminating the need for high temperatures or pressures. This process selectively produces nitrogen oxides (N_*x*_O_*y*_), including NO, NO_2_, and N_2_O_5_, which are soluble in water and readily convert into accessible nitrogen compounds, including nitrite (NO_2_^–^), nitrate (NO_3_^–^), and their conjugate acids, i.e., nitrous acid (HNO_2_) and nitric acid (HNO_3_).^6^ While dielectric barrier discharge systems have been most extensively studied for this purpose, recent efforts have diversified toward other plasma configurations with distinct discharge characteristics.^7,8^ Microwave and radio frequency plasmas enable more uniform electron heating and volumetric reactions, while atmospheric-pressure plasma jets and gliding arcs provide higher energy densities suitable for continuous nitrogen fixation near ambient conditions. These complementary plasma sources collectively broaden the design space for optimizing energy efficiency and product selectivity in plasma-assisted nitrogen fixation. The resulting solution, commonly termed plasma-treated water (PTW),^9^ has gained recognition as a novel, decentralized nitrogen source.

Because PTW is characterized by a cocktail of reactive oxygen and nitrogen species (RONS), it has also attracted interdisciplinary attention. Active attempts have already been made to prove its multifunctional properties across fields such as biomedicine, agriculture, and the food industry.^10,11^ The effectiveness of PTW is largely determined by the composition and concentration of RONS therein, which are in turn affected by the plasma operating conditions. Previous studies have focused mostly on discharge parameters such as the electrode structure,^12^ voltage,^13^ and gas composition^14^ but have relied on posttreatment analysis, providing limited insight into how RONS in PTW evolve during plasma reactions in real time.

To better optimize the PTW composition for specific functions and applications, understanding how external (optional) process variables—beyond the plasma itself—affect RONS formation via real-time measurements is valuable. Two particularly practical variables are the water temperature and stirring rate, yet their effects remain poorly quantified. Shen et al.^15^ studied postdischarge PTW stored at different temperatures, but the influence of the processing temperature remains unknown. Chiappim et al.^16^ compared the pH and oxidation–reduction potential (ORP) of stirred and stagnant PTW at a single stirring speed without exploring the impact of different stirring rates.

In this study, we systematically investigated how the water temperature and stirring speed govern RONS formation in PTW. Plasma treatments were carried out using a surface dielectric barrier discharge (sDBD) reactor under various thermal and stirring conditions. In situ, real-time monitoring of gas-phase species (O_3_ and NO_*x*_) and their aqueous resultants (NO_2_^–^, NO_3_^–^, and HNO_2_) in PTW is conducted using optical absorption spectroscopy (OAS). Our results show that elevated water temperatures prolong an O_3_-dominant regime, promoting NO_3_^–^ formation while suppressing NO_2_^–^ and HNO_2_ formation. Insufficient mixing limits species transport and reaction rates, whereas controlled stirring improves the uniform distribution of species and further increases NO_3_^–^ accumulation. This work introduces a real-time, dual-phase diagnostic approach that simultaneously tracks gas- and liquid-phase chemistry without sampling. By coupling synchronized broadband OAS with advanced spectral deconvolution, this method captures transient O_3_–NO_*x*_ dynamics and their dissolution into the liquid in situ. Unlike previous studies that focused solely on static plasma conditions, this study applies this real-time monitoring approach to systematically evaluate how water temperature and stirring speed govern plasma–liquid reactions. These findings provide insight into how such process parameters can be tuned to tailor the PTW composition for targeted applications.

## Experimental Section

### Experimental Setup

The plasma reactor used in this study is schematically illustrated in Figure 1a. An sDBD source, for which details can be found in ref.,^14^ was mounted on the lid of the reactor to indirectly treat the distilled water in the chamber. To ensure complete dissolution of the generated gaseous reactive species (such as O_3_ and NO*_x_*) in the distilled water without any loss to the external environment, the reactor was designed as a batch-type, gastight system. The reactor body was made of polytetrafluoroethylene (PTFE), which has nonreactive surface properties, to minimize surface reactions and thereby make the chemical environment stable. For each treatment, 250 mL of distilled water was poured into the reactor, the total internal volume of which was approximately 815 cm^3^. The gap between the plasma electrode and the water surface was approximately 60 mm. sDBD was generated in ambient air as the working gas, thus eliminating the need for additional gas supplies and pursuing simplicity and cost-effectiveness. Figure S1a illustrates the electrode configuration of the sDBD source, with the detailed construction and operating principles described in our previous papers.^14,17^ A high-voltage power generator (FTLAB, HPI500) was connected to the sDBD source; two bipolar square voltage waveforms 180° out of phase were applied to the respective electrodes. The discharge was operated at a fixed frequency of 50 kHz with a 25% duty cycle. The electrical characteristics of the plasma (Figure S1b) were obtained with a high-voltage probe (Tektronix, P6015A) and a current probe (Pearson Electronics, Model 4936) combined with a digital oscilloscope (Tektronix, MDO34). Specifically, Figure S1c presents the time-averaged power consumption calculated under conditions of a 20 °C water temperature and a 600 rpm stirring speed, showing the values for both the O_3_ and NO*_x_* modes. The slight increase in power consumption during the transition to the NO*_x_* mode was attributed to changes in the gas temperature and constituents of charged particles, leading to an increased discharge current.^17^ The optical characteristics of the plasma were obtained by optical emission spectroscopy (Figure S2). Emission spectra in the 200–1100 nm range were recorded using a spectrometer (Ocean Optics, HR6) operated with Oceanview software. The spectra exhibited characteristic emission bands of molecular nitrogen (N_2_(C–B)), which are typical of air-based surface dielectric barrier discharges. These optical features indicate that vibrationally excited N_2_ species play a dominant role in the plasma chemistry. Consequently, NO*_x_* formation becomes prominent, and O_3_ and long-lived nitrogen oxides further participate in downstream oxidation reactions.

**Figure 1:**
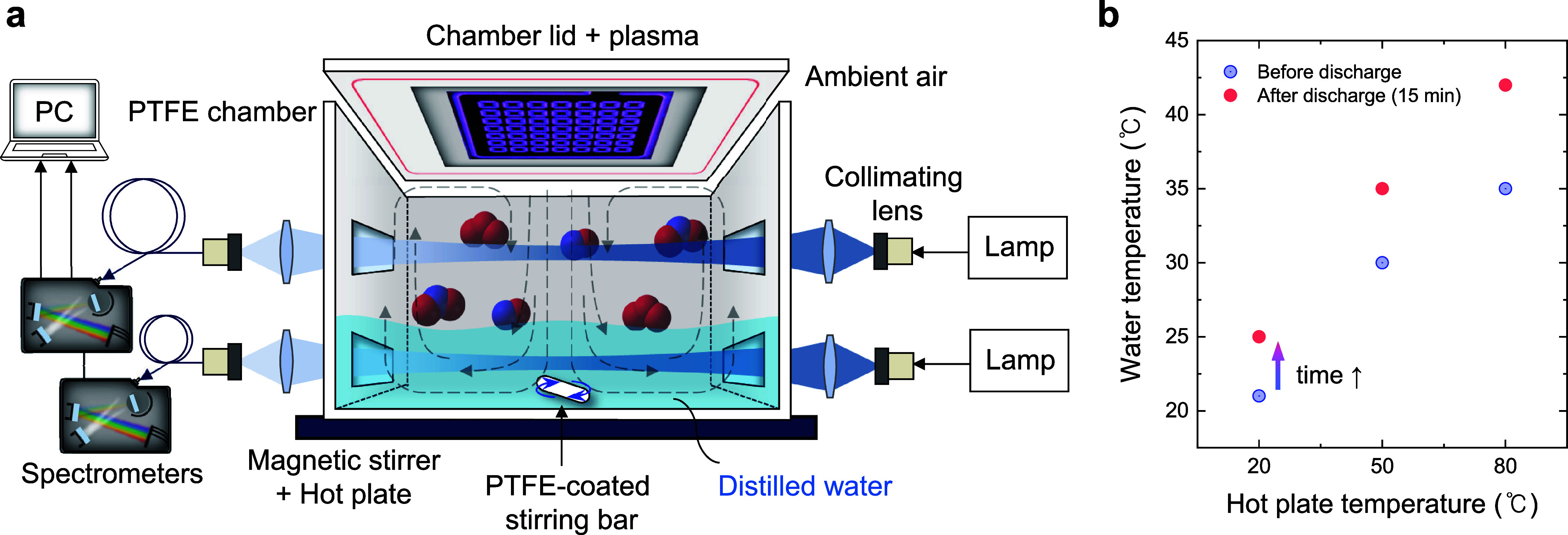
(a) Schematic diagram of the experimental configuration designed to investigate the effect of the water temperature and stirring speed on reactive species formation in PTW. The plasma reactor was equipped with a dual-channel OAS system for real-time measurement of gas- and liquid-phase species. (b) Scatter plot showing the water temperature before and after 15 min of plasma treatment under different hot plate temperatures.

Figure 1a illustrates the experimental setup used to investigate the effect of the water temperature. The reactor was placed on a hot plate (DHIHAN, MSH-20D) to heat the water and maintain set temperatures of 20, 50, and 80 °C. The initial water temperature was verified using a thermometer to determine whether the water temperature had stabilized. During the plasma treatments, the water temperature gradually increased, rising by approximately 4–7 °C after 15 min of plasma exposure (see Figure 1b). To observe the influence of the water temperature, the stirring speed was fixed at an optimal level of 600 rpm during the experiments. A 1 cm-long magnetic stir bar was used while minimizing interference with the absorption measurements. The effect of the stirring speed was examined in the range of 200–1000 rpm. The physicochemical properties of PTW were monitored using a multiparameter meter (Orion Star A215, Thermo Scientific). The complete data set is provided in Table S1 and includes pH, conductivity, ORP, and solution temperature. These parameters and their time-dependent changes were measured under representative treatment conditions. To maintain consistency, the sDBD plasma electrode was not replaced during the experiments, and all the plasma driving conditions except for the stirring speed and water temperature were kept constant throughout the experiment.

The plasma reactor was equipped with two identical broadband OAS setups to simultaneously measure the concentrations of chemical species in both the gas and liquid phases. For each setup, a combination of optical fibers (Ocean Optics, QP400-2-SR) and collimating lenses (Ocean Optics, 74-UV) was used to transmit UV–visible light from a deuterium lamp (Ocean Optics, DH-2000-BAL) through the plasma reactor to the spectrometer (Ocean Optics, Maya2000 Pro). For gas-phase measurements, the spectrometer covered a spectral range of 188–411 nm, with a 25 μm slit width and a 600 lines mm^–1^ diffraction grating. For liquid-phase measurements, a spectrometer with a 5 μm slit width and a 1200 lines mm^–1^ diffraction grating, covering the 199–425 nm range, was employed. Spectral data were automatically collected every second, with averaging of four 250 ms integrated spectra, and all the measurements were repeated three times. The methods used for data analysis are discussed in the following section.

### Data Analysis of OAS Measurements

The number densities of RONS in the gas phase and the molar concentrations of RONS in the liquid phase were both obtained by applying nonnegative least-squares (NNLS) regression to the measured absorption spectra.^18^ In the gas phase, the experimental optical depth spectra τ_ex_ were calculated as _τex_(λ) = ln­[*I*_0_(λ)/*I*(λ)], where *I*_0_(λ) and *I*(λ) are the intensities of the light passing through the reactor in the absence and presence of absorbing RONS, respectively, which were acquired by the spectrometer. The specific data processing method used to find well-fitted synthetic spectra and obtain the corresponding absolute densities of interest was described in our previous papers.^14,17^ The spectra were fitted using the absorption cross sections σ*_j_* of seven species (see Figure S3a) provided by the MPI-Mainz UV/vis Spectral Atlas:^19^ O_3_,^20^ NO,^21^ NO_2_,^22^ N_2_O_4_,^22^ N_2_O_5_,^23^ HONO,^23^ and HONO_2_.^23^

The number densities of the fitted species were determined using the Beer–Lambert law, $\tau_{\mathrm{ex}}\left(\lambda \right)=\underset{j}{\sum }\sigma_{j}\left(\lambda \right)n_{j}l_{G}+ο\left(\lambda \right)$ , where *n_j_* represents the number density of the *j*-th RONS, *l* is the gas absorption path length, which was set at 15 cm in this study, and *ο*(λ) is experimental noise. When we attempted to find well-fitted synthetic spectra for deconvolution, an example of which is shown in Figure 2a for an absorption spectrum measured at 90 s, the corresponding least-squares fitting clearly demonstrated that erroneous fitting can occur with unnecessary curves for some absorbing species. The least-squares fitting algorithm occasionally included HONO_2_ in the synthetic spectra in an attempt to replicate the measurement noise near 210 nm. In addition, NO_2_ and HONO seemed to be overestimated, possibly to reconstruct the noise-level baseline above 325 nm because linear regression naturally prioritizes the accuracy of spectral fitting at high measured optical depth values, leading to overestimation of NO_2_ and HONO. Thus, based on a heuristic post-processing method (reported in our previous paper),^18^ such erroneous fitting was avoided, and some unavoidable erroneously estimated number densities were removed from the time series data (Figure 2b). Because each measured optical depth spectrum was acquired over 1 s, the number density time series of the gas-phase RONS was plotted at 1 s intervals. Afterward, the noise in the raw time series data was cleaned by first zeroing densities smaller than the respective limit of detection for each species and then replacing briefly appearing or disappearing densities with linear interpolation of their neighboring values. In this study, nonzero densities or zero densities persisting for less than 4 s were considered noise and were cleaned.

**Figure 2:**
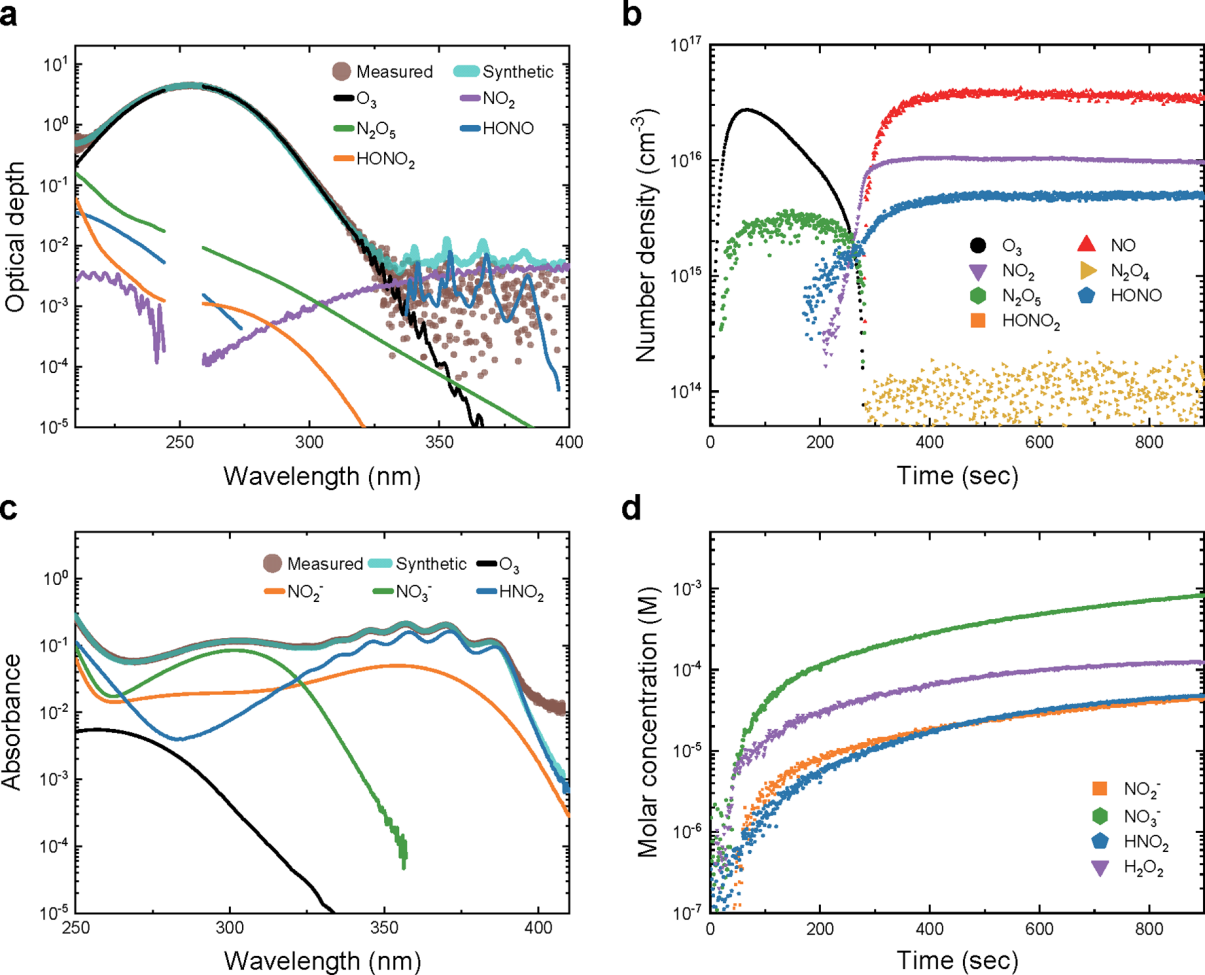
OAS analysis and data processing for gas-phase and liquid-phase RONS. (a) Example of an optical depth spectrum at 90 s, showing measured data and the fitted contributions of gas-phase RONS. HONO_2_ is overestimated due to noise near 210 nm, while HONO and NO_2_ are overfitted in the 340–400 nm range, where their contributions exceed the measured optical depth. (b) Final number density time series data after removal of HONO_2_ noise and HONO and NO_2_ overestimation. (c) Broadband nonnegative linear regression deconvolution across the 250–410 nm measured absorbance data, assuming spatially homogeneous RONS molar concentrations along the absorption path. (d) Final molar concentration time series data derived from the spectral analysis of each recorded absorbance spectrum.

The experimental absorbance spectra *A*_ex_ in the liquid phase were calculated using the equation *A*_ex_(λ) = log_10_[*I*_0_(λ)/*I*(λ)]. The measured absorbance spectra from 250.00 to 409.98 nm were fitted using the molar absorption coefficients ε*_j_* of five species, O_3_,^24^ NO_2_^–^, NO_3_^–^, HNO_2_, and H_2_O_2_, as provided in Figure S3b. Except for O_3_, the molar absorption coefficients were determined in this study; the corresponding absorption spectra and standard curves are given in Figure S4. After spectral fitting of the liquid-phase RONS (Figure 2c), the molar concentrations of the fitted species were recovered using the Beer–Lambert law, $A_{\mathrm{ex}}\left(\lambda \right)=\underset{j}{\sum }\varepsilon_{j}\left(\lambda \right)c_{j}l_{L}+ο\left(\lambda \right)$ , where *c_j_* is the molar concentration of the *j*-th RONS and *l*_L_ is the liquid absorption path length of 15 cm. Each measured absorbance spectrum was measured with an exposure time of 1 s; the time series molar concentration of the liquid-phase RONS was plotted at 1 s intervals (see Figure 2d).

## Results and Discussion

Figure 3 presents the temporal evolution of the concentrations of reactive species in both the gas phase (top) and liquid phase (bottom), highlighting the impact of the water temperature on them. This case experiment was conducted with a constant stirring speed of 600 rpm to reveal the temperature effects while maintaining consistent gas–liquid mass transfer. At the onset of plasma discharge, ozone (O_3_) was initially the predominant species under all conditions. Over time, nitrogen oxides (NO*_x_*) gradually formed, leading to a transition from an O_3_-dominated to a NO*_x_*-dominated chemistry, a process that has been extensively discussed in previous studies.^25,26^ In addition to O_3_, short-lived reactive oxygen species such as hydroxyl radicals (•OH) and singlet oxygen (^1^O_2_) may also play a transient role in nitrogen oxidation within the plasma phase. Sakiyama et al.^27^ have shown that these radicals are generated within microseconds after breakdown and can momentarily and periodically interact with NO and NO_2_, thereby contributing to the early stages of plasma-induced nitrogen oxidation. Their high reaction rate constants (≈10^–11^ to 10^–12^ cm^3^ s^–1^) suggest that these species influence the nitrogen oxidation kinetics before O_3_ and long-lived NO_*x*_ species become dominant in the gas chemistry.^28^

**Figure 3:**
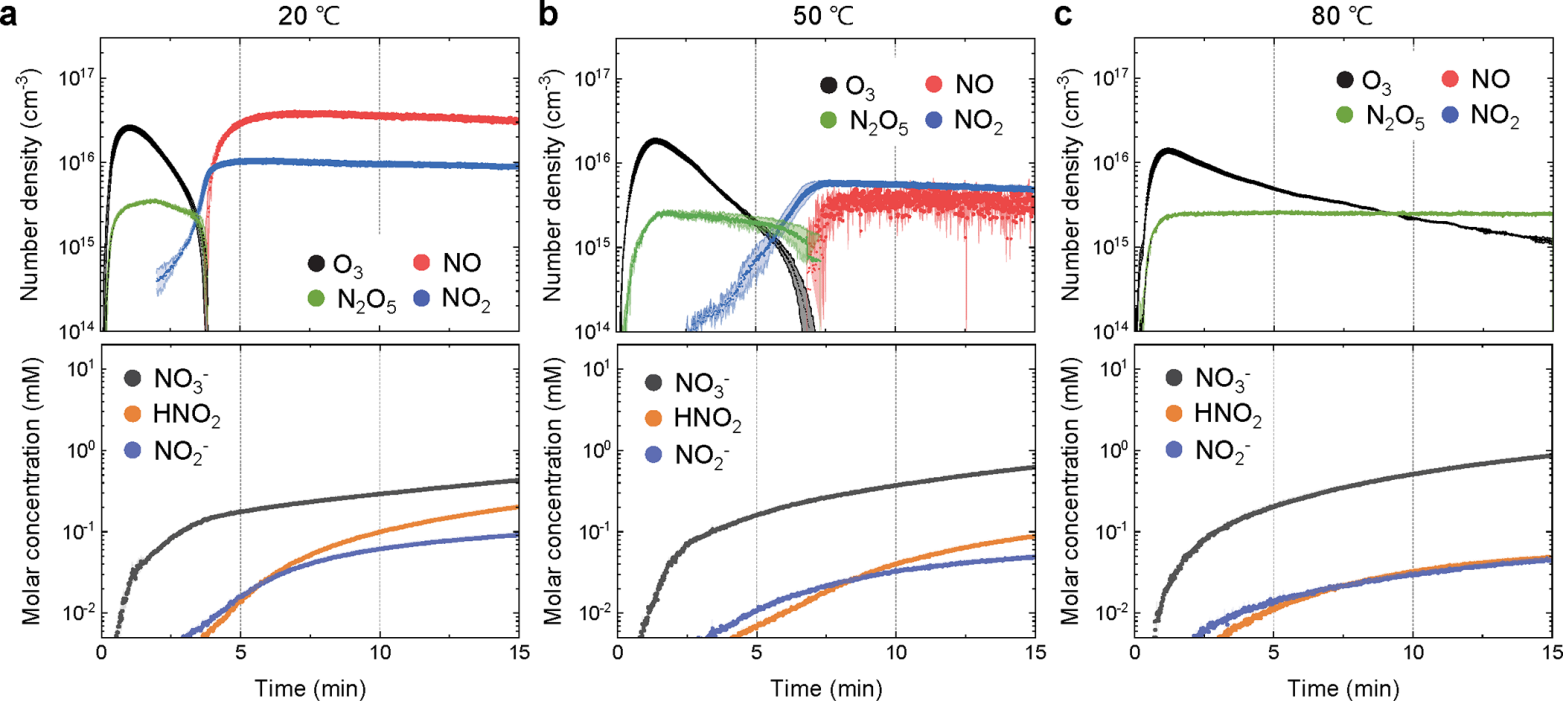
Time-dependent behavior of key reactive species in both the gas phase (top graphs) and liquid phase (bottom graphs) of PTW under various water temperature conditions: (a) 20 °C, (b) 50 °C, and (c) 80 °C (based on the hot plate settings). Gas-phase data include O_3_ (black), NO (red), NO_2_ (blue), and N_2_O_5_ (green), while liquid-phase data include the concentrations of NO_3_^–^ (dark gray), HNO_2_ (blue), and NO_2_^–^ (orange). The time axes in the upper and lower graphs are aligned to illustrate the synchronized evolution of gaseous and aqueous species.

At 20 °C, this transition occurred at approximately 218 s (see Figure 3a). In contrast to the results in ref.,^25,26^ as the water temperature increased, the transition was significantly delayed. Notably, at 80 °C, the reactor maintained the O_3_ mode throughout the entire plasma treatment period (Figure 3c). This delay in mode transition can be attributed to the increased humidity within the plasma chamber caused by the elevated water temperature. According to previous studies, while increased humidity has been found not to significantly impact the total power consumption,^29^ it plays a critical role in altering the chemical reaction pathways and reaction rates in the gas phase. In other words, the presence of water vapor in the discharge gap between the electrode and the water surface may facilitate or hinder a range of chemical reactions. As previously mentioned, humidity accelerates the vibrational-to-translational relaxation rates of N_2_ and O_2_,^30^ leading to a decrease in the atomic oxygen (O) density.^31^ Additionally, as the reaction O­(^1^D) + H_2_O → 2OH is enhanced, the amount of hydroxyl radical in the reactor becomes sufficient to subsequently react with atomic oxygen, forming hydroperoxyl radicals (HO_2_). These radicals compete with O_3_ formation pathways, ultimately lowering the maximum O_3_ concentration.

Moreover, under high humidity conditions, a decreased O_3_ concentration restricts NO_2_ formation. NO production is similarly affected due to the suppression of the Zel’dovich mechanism (O + N_2_(v) → NO + N) in humid environments.^32^ As a result, at 80 °C, neither NO nor NO_2_ was detected during the 15 min plasma treatment, and the reactor remained in the O_3_ mode throughout. These results indicate that the relative balance between H_2_O and NO_*x*_ inside the reactor governs the dominant plasma reaction pathways. When the H_2_O fraction increases, enhanced quenching suppresses NO-forming reactions, thereby reducing O_3_ consumption and shifting the overall reaction kinetics toward oxidation-dominant routes.^33^ As a result, the selectivity of plasma-driven nitrogen fixation changes, favoring the formation of higher oxidation products such as NO_3_^–^. These results demonstrate that variations in the water temperature directly affect the gas-phase plasma chemistry, subsequently altering the chemical composition of the treated liquid.

As shown in the lower graphs in Figure 3, the total concentration of NO_3_^–^ in PTW gradually increased with increasing water temperature. Specifically, at 80 °C, the final NO_3_^–^ concentration reached 0.86 mM, whereas it was 0.43 mM at 20 °C, representing an approximately 2-fold increase. Similarly, NO_2_^–^ and HNO_2_ had increasing trends for all temperature conditions over the treatment time but were more affected by temperature than NO_3_^–^.

At 80 °C, the abundant presence of NO_3_^–^ and the NO_2_^–^-to-HNO_2_ ratio indicated a significant pH reduction from 7 to nearly the p*K*_a_ value of HNO_2_, which subsequently slowed NO_2_^–^ formation as follows: the lower pH of PTW shifted the equilibrium of NO_2_^–^ + H^+^ ⇌ HNO_2_ toward HNO_2_, and subsequently, HNO_2_ underwent oxidation, leading to rapid conversion into NO_3_^–^. Thus, with increasing water temperature, the prolonged O_3_ mode resulted in greater accumulation of NO_3_^–^, while the formation of NO_2_^–^ and HNO_2_ was suppressed.

This trend can also be attributed to the temperature dependence of the reaction kinetics. Generally, higher temperatures increase the reaction rates, as described by the Arrhenius equation $\left(k=A\;e^{-E_{a}/RT}\right)$ , where *k* is the rate constant, *A* is the preexponential factor specific to each reaction, *E*_a_ is the activation energy, *R* is the gas constant, and *T* is the absolute temperature. However, thermodynamically unstable reactive nitrogen species, such as NO_2_^–^ and HNO_2_, may exhibit anti-Arrhenius behavior due to their negative activation energy.^34^ As a result, their production rates decrease with increasing temperature, making them more prone to rapid decomposition and oxidation into NO_3_^–^. Ultimately, higher water temperatures promote NO_3_^–^ as the dominant species in PTW, highlighting the role of temperature as a critical control parameter for modulating the chemical composition of PTW.

Fina et al.^35^ also reported that mild heating of PTW significantly enhanced its bactericidal efficacy against *E. coli*, suggesting that temperature-induced changes in the chemical composition may contribute to synergistic antimicrobial effects. Similarly, the use of PTW as a potential method for sanitizing eggshells has been explored.^36^ However, although maintaining microbial safety without damaging the eggshell cuticle is essential, optimization of the plasma treatment at typical egg washing temperatures (40–46 °C) remains limited. This highlights the importance of understanding how elevated water temperatures affect the PTW composition in industrial applications. These findings underscore the potential of temperature regulation as a strategic approach for optimizing and maintaining the desired chemical profile of PTW in practical applications.

In the following, for the same procedure, the variations in the gas-phase and liquid-phase reactive species concentrations with the stirring speed are discussed. No significant changes in the O_3_ and NO_*x*_ concentrations were induced by different stirring speeds at 20 °C, demonstrating that the O_3_–NO_*x*_ mode transition was almost consistent across all stirring conditions (Figure 4). The transition time was maintained at approximately 4 min, irrespective of the stirring speed. This result suggests that the gas-phase mode transition is primarily determined by plasma reactions, with a negligible impact from stirring.

**Figure 4:**
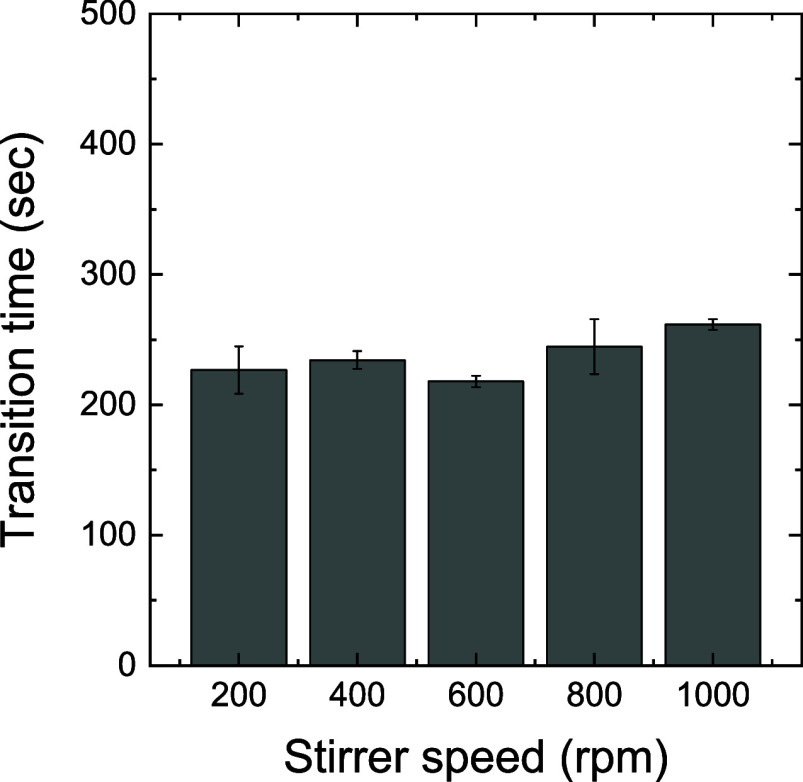
Transition times from O_3_ to NO_*x*_ in the reactor under various stirring speeds, 200–1000 rpm, at the fixed temperature of 20 °C. Raw gas- and liquid-phase data for each stirring speed are provided in Figure S5. Note that the transition time was determined as the mean time at which the ozone concentration dropped to below 10^15^ cm^–3^.

However, depending on the reactor design, stirring possibly influences gas-phase reactions. Berlatto et al.^37^ reported that stirring modifies the discharge characteristics in configurations where the electrodes and liquid are in close proximity or in direct contact. Our sDBD reactor was designed to ensure the optical path and thereby allow synchronized in situ OAS while minimizing perturbations to the sDBD.

In this context, the primary role of stirring lies in enhancing the mass transfer at the gas–liquid interface.^38^ In an indirect treatment configuration, long-lived gas-phase species such as O_3_, NO, NO_2_, and N_2_O_5_ are the dominant contributors to the resulting liquid-phase chemistry.^39^ Stirring facilitates dissolution and uniform distribution of these species within the liquid. This effect becomes particularly significant in gastight reactors where natural convection is absent. Our approach therefore emphasizes the use of stirring not to modulate gas-phase plasma reactions but rather to improve species transport and ensure homogeneity in the liquid phase.

Figure 5a, b, and c presents the real-time variations in NO_3_^–^, HNO_2_, and NO_2_^–^ in PTW, respectively. The results reveal that their formation in PTW can be effectively regulated by stirring, which changes the mass transfer and reactivity, underscoring the energy-efficient role of stirring in PTW processing. At stirring speeds of 800 rpm or higher, the vortex in the treated water disrupted the optical path for absorption spectroscopy measurements, making real-time monitoring challenging. To overcome this issue, stirring was temporarily paused, and spectral acquisition was performed at 1 min intervals. This adjustment is reflected in the graphs, where the individual data points are relatively sparse for the two cases.

**Figure 5:**
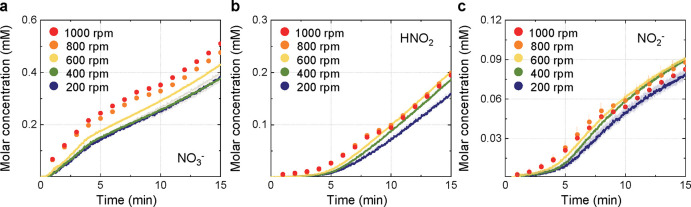
Time-dependent concentrations of (a) NO_3_^–^, (b) HNO_2_, and (c) NO_2_^–^ in PTW under various stirring speeds (200–1000 rpm). Each graph presents the concentration profile of a specific species obtained from the same set of experiments. The scatter plots for 800 and 1000 rpm indicate measurements done at 1 min intervals. All data points represent the mean values along with standard deviations from three independent replicates under identical experimental conditions.

Figure 5a shows that NO_3_^–^, which is the most thermodynamically stable species in PTW, exhibited a continuous increase in its concentration with increasing stirring speed. At 200 rpm, the final NO_3_^–^ concentration was approximately 0.383 mM, whereas it increased to 0.510 mM at 1000 rpm, corresponding to an approximately 33% increase. This gradual increase is attributed to the high solubility of HNO_3_, from which NO_3_^–^ predominantly originates. HNO_3_, being a strong acid, has a high Henry’s constant (5.1 × 10^6^),^39^ enabling it to readily dissolve in the aqueous phase. Stirring enhances the gas–liquid mass transfer coefficient, thereby promoting HNO_3_ dissolution and facilitating accumulation of NO_3_^–^. Consequently, the increase in the NO_3_^–^ concentration was proportional to the increase in the stirring speed up to 1000 rpm, indicating a zero-order kinetic trend.^37^ This observation offers a practical strategy to simply modulate NO_3_^–^ formation in PTW by stirring.

At the beginning of the treatment, the variations in the formation rates of both HNO_2_ and NO_2_^–^ species, which become faster within 5 min, differed from those of NO_3_^–^ (see Figure 5b, c). As the stirring speed increased to 800 and 1000 rpm, their formation began to decline after the 10 min mark. This decline can be attributed to excessive stirring facilitating volatilization of dissolved gas-phase reactive species, thereby reducing their stability in the liquid phase.^40^ In particular, HNO_2_, which has a relatively low Henry’s constant (1198) compared to HNO_3_,^39^ is more susceptible to volatilization losses under vigorous agitation. Additionally, as the pH decreases during treatment, the equilibrium NO_2_^–^ ⇌ HNO_2_ shifts toward HNO_2_, which is thermodynamically unstable and gradually undergoes self-oxidation or disproportionation (3HNO_2_ ↔ H^+^ + NO_3_^–^ + 2NO + H_2_O; HNO_2_ + 3H_2_O_2_ → 2HNO_3_ + H_2_O).^41,42^ Under vigorous mixing, this oxidative conversion of transient HNO_2_/NO_2_^–^ species into the more stable NO_3_^–^ is further promoted. These findings highlight the dual role of stirring in PTW chemistry. Sufficient stirring enhances the gas–liquid mass transfer coefficient, promoting dissolution and diffusion of reactive species. Convective mixing also strengthens interfacial transport, reducing concentration gradients of key intermediates such as NO, NO_2_, and HNO_2_ near the gas–liquid boundary. As a result, reactive nitrogen species undergo more homogeneous reactions throughout the liquid, leading to a stable and reproducible product composition. It can also be quantitatively interpreted using the classical Sherwood–Reynolds–Schmidt correlation,^43^ which predicts a substantial enhancement of the effective mass transfer coefficient under stirred conditions. These findings demonstrate that hydrodynamic mixing plays a key role in controlling interfacial transport and, consequently, the selectivity of plasma-driven nitrogen fixation. In contrast, excessive stirring favors volatilization and further oxidation, hindering accumulation of less stable species such as HNO_2_ and NO_2_^–^, whereas insufficient stirring limits mass transfer and reduces the reaction efficiency.

Notably, both the HNO_2_ and NO_2_^–^ concentrations increased around the 4 min mark, coinciding with the transition from the O_3_-dominant to the NO_*x*_-dominant regime in the gas phase, as shown in our previous study.^14^ This result suggests that the gas-phase chemistry directly influences the liquid-phase reaction pathways. Once NO and NO_2_ are abundantly produced, their dissolution drives the formation of HNO_2_ and NO_2_^–^ in the solution. Therefore, the generation of these nitrogen species is governed by both the availability of gaseous precursors and the stirring conditions that control their dissolution and transport. Precise regulation of stirring is thus essential for optimizing the PTW composition, ensuring consistency, and enabling effective process control.

## Conclusions

This work clarifies how auxiliary hydrodynamic and thermal parameters govern the gas–liquid transfer of plasma-generated nitrogen oxides in PTW. Figure 6 summarizes the terminal concentrations of the “nitrite pool” (HNO_2_ + NO_2_^–^) and nitrate (NO_3_^–^) after 15 min of treatment while the sDBD conditions were held constant. The data show that simple tuning of the stirring rate and bulk-water temperature allows the reactive-nitrogen speciation in PTW to be tailored on demand. Stirring has proven to be an effective strategy for enhancing mass transfer and ensuring uniform distribution of reactive species without additional plasma power consumption. The optimal stirring speed facilitates efficient mixing while preserving the plasma operation stability. Nitrate, which is the most stable species in PTW, exhibited a steady increase in its concentration with the stirring speed, reaching its peak at 1000 rpm, whereas a moderate rate (600 rpm) maximized HNO_2_ + NO_2_^–^ by homogenizing the liquid while limiting volatilization losses. Increasing the water temperature modulated the plasma chemistry by increasing the humidity, thereby prolonging the O_3_-dominant regime and delaying the O_3_ → NO_*x*_ transition. The result was selective enhancement of NO_3_^–^ formation concurrent with suppression of HNO_2_ + NO_2_^–^. These real-time observations extend earlier O_3_/NO_*x*_ transition studies conducted in controlled N_2_/O_2_ mixtures to more practical cases of ambient-air plasmas.

**Figure 6:**
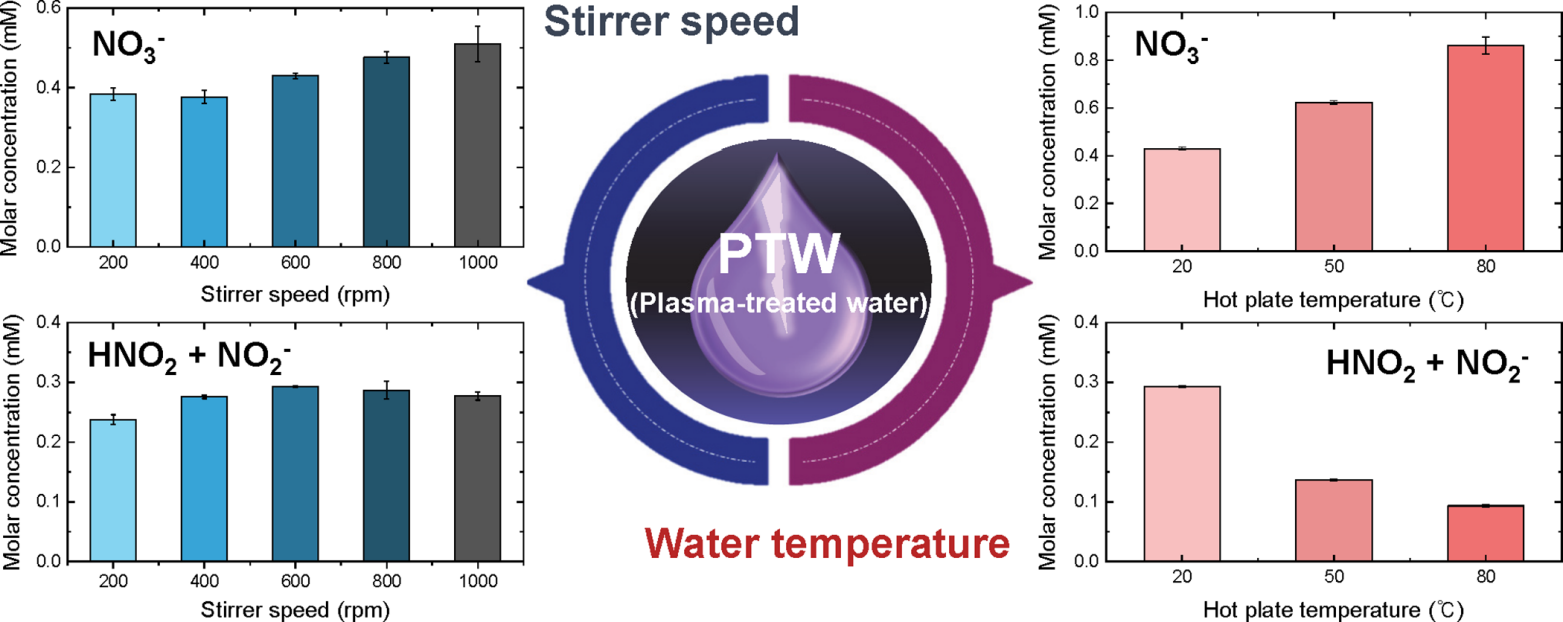
Summary of the final concentrations of major nitrogen-based species in PTW after 15 min of plasma treatment under different process parameters. The left panels show the effect of the stirring speed (200–1000 rpm), while the right panels present the effect of the water temperature (20–80 °C). This figure provides a comparative overview of the influence of each parameter on the species production trends.

Therefore, precise control over the stirring speed and temperature is critical for maximizing the reaction efficiency, stabilizing product speciation, and expanding the functional scope of PTW in environmental and industrial applications. Although this study was conducted under gastight batch conditions to isolate the effects of temperature and stirring, the observed mechanistic trends are expected to remain qualitatively valid in open or continuous-flow plasma–liquid systems. In open configurations, humidity buildup and gas residence time differ substantially, which can quantitatively alter the O_3_/NO_*x*_ ratio and product distribution. For instance, Chiappim et al.^16^ demonstrated in an open gliding-arc system that magnetic stirring improved mass transfer, homogenized pH and ORP, and enhanced plasma–liquid reactivity. These findings suggest that although absolute concentrations may vary depending on reactor openness and flow dynamics, the fundamental dependencies of reactive species behavior on temperature and hydrodynamic mixing remain robust. This study thus establishes a mechanistic framework under well-controlled batch conditions and provides a valuable foundation for extending plasma-driven nitrogen fixation toward scalable, flow-through configurations.

## Supplementary Material



## Notes

Characteristic of the surface dielectric barrier discharge (sDBD) plasma source; absorption cross sections and molar absorption coefficients; UV–vis absorbance spectra and corresponding calibration curves; time-dependent concentrations of raw data under all conditions (PDF)
