# Supplementary material for: Effect of Process Parameters on HNO y=2,3 and NO y – Formation in Plasma-Treated Water
Source: ACS Omega. 2025 Dec 2;10(49):60332–40. doi: 10.1021/acsomega.5c07020 (PMC12713498; doi:10.1021/acsomega.5c07020)
Supplement: Supplementary file 1 [file ao5c07020_si_001.pdf]

Supporting Information

Effect of Process Parameters on $HNO_{y=2,3}$ and NO_y^- Formation in Plasma-Treated Water

Jin Hee Bae[1], Seong-Cheol Huh[1], Negar Rahdar[1], Hyungyu Lee[2] and Sanghoo Park[1,*]

[1]Department of Nuclear and Quantum Engineering, Korea Advanced Institute of Science and Technology, 291 Daehak-ro, Yuseong-gu, Daejeon 34141, Republic of Korea
[2]Department of Electronic and Biological Physics, Kwangwoon University, 20 Gwangun-ro, Nowon-gu, Seoul 01897, Republic of Korea

* Corresponding author, E-mail address: sanghoopark@kaist.ac.kr (S. Park).

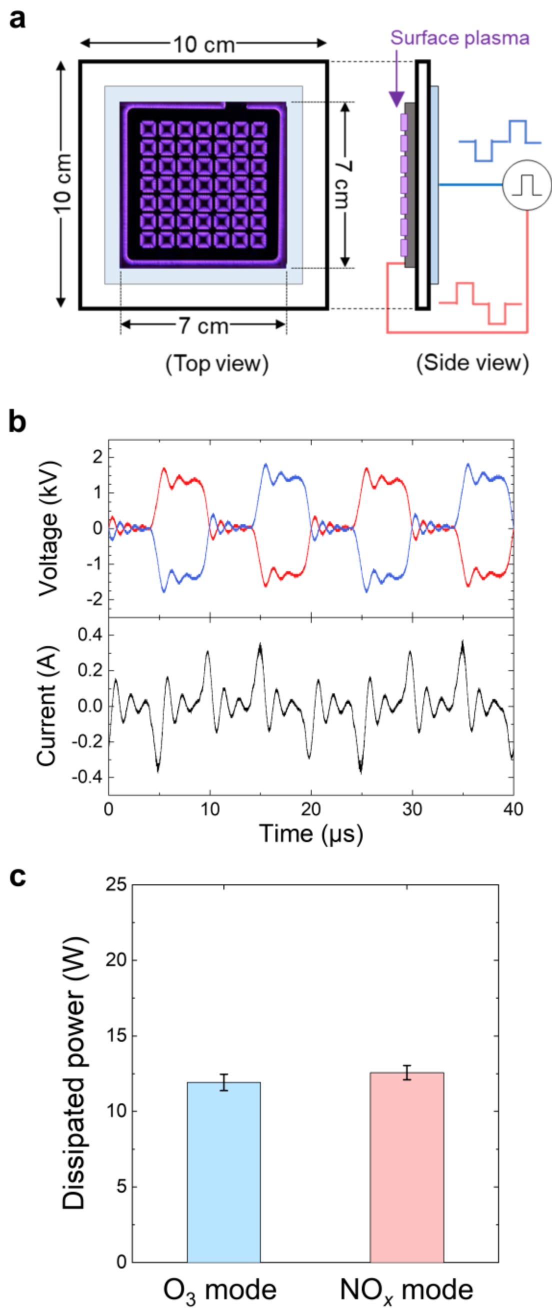

Figure S1. Characteristic of the surface dielectric barrier discharge (sDBD) plasma source. (a) Configuration of the sDBD electrode structure used in this study, along with the applied waveform illustrating oppositely phased signals on each electrode. (b) Voltage–current waveform applied to the sDBD plasma source. (c) Cycle-averaged power dissipated under O_3 and NO_x chemical modes at a water temperature of 20 °C.

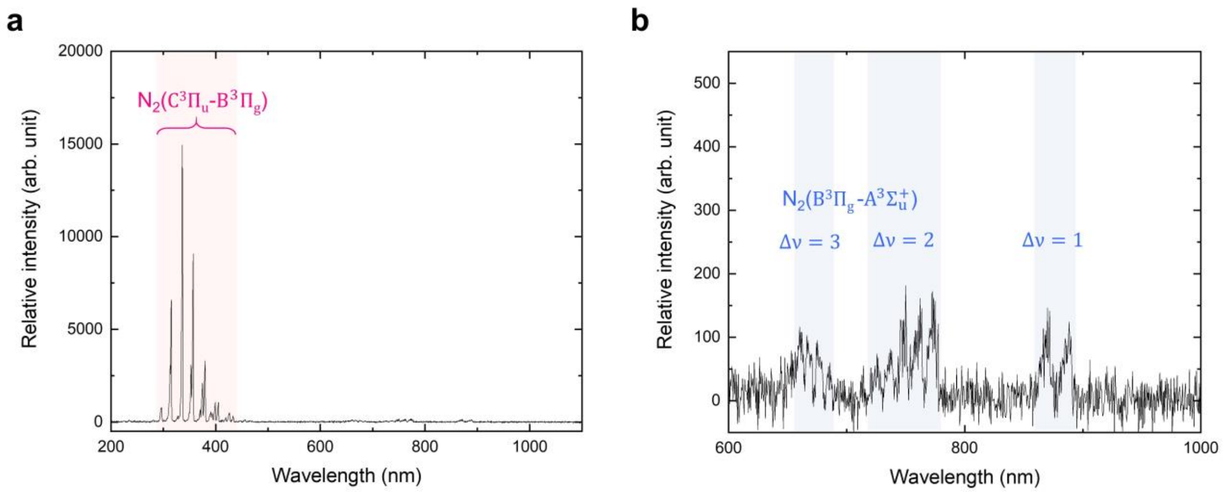

Figure S2. Optical emission spectroscopy of the sDBD plasma source operated in ambient air. (a) Full emission spectrum recorded in the 200–1100 nm range using an Ocean Optics HR6 spectrometer. The main peak corresponding to the second-order positive system $N_2(C^3\Pi_u \rightarrow B^3\Pi_g)$ is clearly observed. (b) Expanded view of the 600–1000 nm region from (a), highlighting the $N_2(B^3\Pi_g \rightarrow A^3\Sigma_u^+)$ first positive system observed in the near-infrared range.

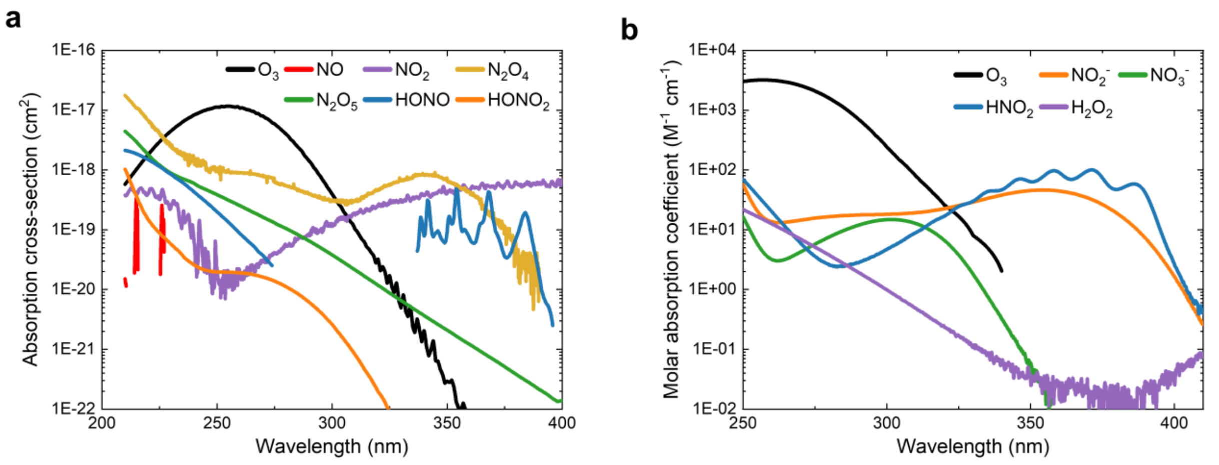

Figure S3. The absorption cross-sections and molar absorption coefficients used for analysis in this study. (a) Absorption cross-sections of O_3, NO, NO_2, N_2O_4, N_2O_5, HONO, and $HONO_2$ in 210–400 nm. (b) Molar absorption coefficients of O_3, NO_2^-, NO_3^-, HNO_2, and H_2O_2 in 250–410 nm.

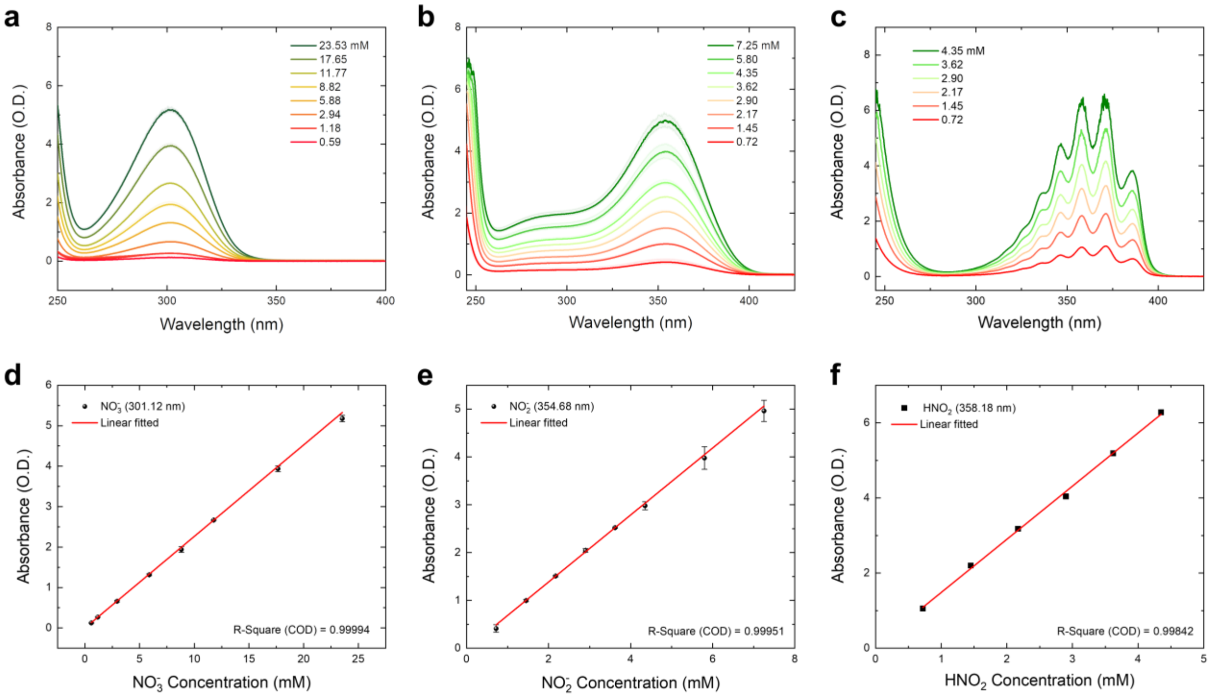

Figure S4. UV–Vis absorbance spectra and corresponding calibration curves for standard solutions of nitrate (NO_3^-), nitrite (NO_2^-), and nitrous acid (HNO_2). (a–c) Absorbance spectra of NO_3^-, NO_2^-, and HNO_2 at varying concentrations, respectively. (d–f) Calibration curves for NO_3^-, NO_2^-, and HNO_2 constructed from the spectral data in (a), (b), and (c), respectively. All measurements were performed in triplicate to ensure reproducibility, and the error bars represent standard deviations.

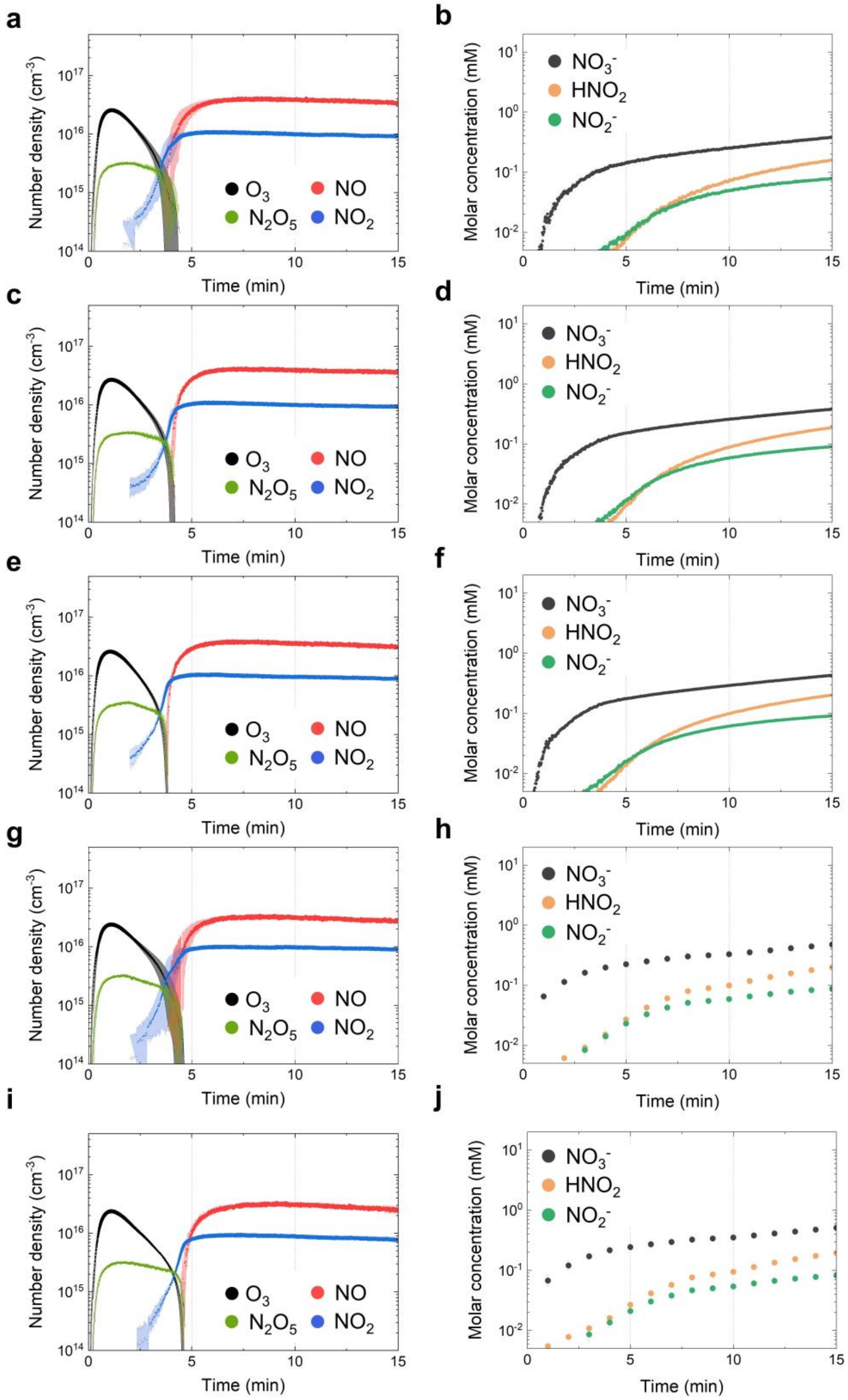

Figure S5. Time-dependent concentrations of gas-phase and liquid-phase reactive species in PTW under different stirring speeds. (a, b) Gas-phase (O_3, NO, NO_2) and corresponding liquid-phase (NO_3^-, HNO_2,

NO_2^-) data at 200 rpm; (c, d) at 400 rpm; (e, f) at 600 rpm; (g, h) at 800 rpm; (i, j) at 1000 rpm. Left column: gas-phase number densities; right column: corresponding liquid-phase concentrations. All measurements were conducted using synchronized optical absorption spectroscopy to capture real-time species evolution in both phases.

Table S1. Physicochemical properties of plasma-treated water (pH, conductivity, oxidation–reduction potential (ORP), and solution temperature) were measured after 5, 10, and 15 min of plasma treatment under representative experimental conditions. Note that the data for 600 rpm and 20 °C conditions are identical, and are presented in both cases for ease of comparison.

	pH	Conductivity (μS/cm)	ORP (mV)	Temperature (°C)
Control	5.71 ± 0.07	4.98 ±0.26	41.9 ± 4.31	21.23 ± 0.04
200 rpm (5 min)	3.27 ± 0.01	501.46 ± 2.70	182.4 ± 0.37	24.33 ± 0.24
200 rpm (10 min)	2.99 ± 0.04	695.26 ± 1.68	191.23 ± 0.61	25.6 ± 0.21
200 rpm (15 min)	2.66 ± 0.02	779.23 ± 2.74	198.23 ± 0.20	27.3 ± 0.04
600 rpm (5 min)	3.27 ± 0.01	522.13 ± 2.30	183.23 ± 0.28	24.46 ± 0.26
600 rpm (10 min)	3.05 ± 0.02	796.26 ± 7.95	196.9 ± 0.29	25.7 ± 0.14
600 rpm (15 min)	2.60 ± 0.09	992 ± 16.75	199.1 ± 1.84	27.0 ± 0.16
1000 rpm (5 min)	3.22 ± 0.08	505.43 ± 5.14	185.4 ± 0.57	24.4 ± 0.21
1000 rpm (10 min)	2.91 ± 0.08	699.8 ± 6.07	199.73 ± 0.12	25.1 ± 0.08
1000 rpm (15 min)	2.67 ± 0.08	989.66 ± 8.33	200.16 ± 0.20	26.63 ± 0.12
20 ℃ (5 min)	3.27 ± 0.01	522.13 ± 2.30	183.23 ± 0.28	24.46 ± 0.26
20 ℃ (10 min)	3.05 ± 0.02	796.26 ± 7.95	196.9 ± 0.29	25.7 ± 0.14
20 ℃ (15 min)	2.60 ± 0.09	992 ± 16.75	199.1 ± 1.84	27.0 ± 0.16
50 ℃ (5 min)	2.89 ± 0.04	619.63 ± 1.14	188.56 ± 0.32	32.46 ± 0.28
50 ℃ (10 min)	2.76 ± 0.03	731.3 ± 0.82	200.46 ± 1.34	34.23 ± 0.20
50 ℃ (15 min)	2.58 ± 0.03	928.6 ± 2.35	207.1 ± 1.56	35.26 ± 0.20
80 ℃ (5 min)	2.77 ± 0.02	633.6 ± 13.38	189.96 ± 0.33	36.46 ± 0.20
80 ℃ (10 min)	2.49 ± 0.01	753.8 ± 1.15	213.2 ± 0.53	39.23 ± 0.68
80 ℃ (15 min)	2.40 ± 0.02	933.73 ± 3.53	222.73 ± 0.28	42.26 ± 0.20
